# Necrology

**Published:** 1894-05

**Authors:** 


### Necrology.

Died at Ann Arbor, Mich., April 14, 1894, of an attack of apoplexy, Dr. Corydon L. Ford, M.D., in the 81st year of his age.

He had for the last few months been in more than usual good health. On the 13th about midday, he started from the Dental College to his home, and while on the way without any premonition he was stricken down; was removed to his home, and died the next morning. He had delivered the last lecture of his course for this year to the medical students the day before the attack. It was remarked by those who heard him that he seemed to have the vigor and energy of younger days. This completed his fortieth year of work in the Medical Department of the University of Michigan. His work here, however, was only a part of the work of his professional life. He began his work as teacher of anatomy in the Medical College at Geneva, New York, in 1842, immediately after his graduation.

During his course of medical study he was a pupil of Dr. E. Carr, of Canandaigua, in the study of dentistry. In this he soon attained such proficiency as enabled him to combine dentistry with such general practice as his health enabled him to engage in, while not on duty as demonstrator of anatomy.

January 25th, 1842, he took his medical degree. He had developed such a taste and aptitude for anatomic pursuits that on the day of his graduation he was appointed Demonstrator of Anatomy in the college at Geneva. He occupied this position till January 1849. In 1846 Buffalo Medical College was organized, when he was called to be Demonstrator of Anatomy in that institution. There he served for three years, when he was appointed Professor of Anatomy in Castleton Medical College, Vermont, where he continued to lecture till 1862. The honorary degree of A.M., was conferred upon him by Middlebury College in 1856.

In 1854 he was appointed Professor of Anatomy in the University of Michigan, where he has continued his annual course of lectures on anatomy and physiology for forty years. In 1860 he was appointed Professor of Anatomy in Berkshire Medical College, Pittsfield, Mass., where he lectured for several years, at the period of the year when the University of Michigan was not in session. In 1864 he accepted the Chair of Anatomy in the Medical School of Maine, Bowdoin College at Brunswick; this position he held till 1870, when he took a recess from his labors in Europe. After which he was appointed to the Chair of Anatomy in Long Island College, Hospital Medical School, Brooklyn, N.Y., which position he held for nineteen years. These courses were given in the intervals of his work in the University of Michigan. Thus it is quite apparent that Dr. Ford's life has been an exceedingly busy one He has delivered on his favorite subject one hundred and nine courses of lectures. During the last third of a century he has been considered one of the best authorities on anatomy in this country. The whole period of his work as teacher of anatomy has occupied fifty-two years, and with unsurpassed success from the beginning to the end; a career very exceptional indeed. Of him it is said, that no man in this country at least has taught anatomy to a greater number of students, and no one in any country has taught better or more faithfully, and inspired more enthusiasm.

In 1878 he was made a member of the Faculty of the Dental Department of the University of Michigan, and has given special instruction to dental students, dwelling at greater length and with more minutiæ on topics that more directly concerned the dental practitioner than any other teacher. In aid of this work, he has prepared questions on anatomy and physiology of the teeth and associated parts for dental students. With a view of awakening an interest in a subject closely allied to the work of the dentist he prepared a syllabus of lectures and questions on Odontology—human and comparative—for the Dental Student.

In 1881 the University of Michigan conferred upon him the title of LL.D. During the years 1852-3, and 1853-4, Dr. Ford was connected with the New York College of Dentistry, at Syracuse, gave instructions in anatomy in that institution, where he received the title of D.D.S. This connection was severed upon his appointment to the University of Michigan in 1854.

Such a life is grand and glorious, full of active, useful work, from the beginning to the end of its career. We can now recall the name of no one in this country who has excelled or even equalled Dr. Ford in all the great work he has accomplished. An almost innumerable host of the medical profession are ready to rise and call him blessed; and may they all seek to emulate the noble example which he has left for them.

Died, at seven o'clock Sunday evening, April 22, at his home in Lexington, Ky., Dr. A. O. Rawls.

The doctor having been indisposed for a time, for relief took chloral, and in such quantity as to produce fatal result. This is a great surprise to all the members of the profession who knew him.

Dr. Rawls was born at Wilmington, Ohio, December 8, 1841. He began the study of dental surgery with Dr. John Doughty, of Connersville, Ind., in 1858. His pupilage continued for two years, when he entered the service of his country, in the Third Indiana Artillery, in June 1861. He served three years, then went to Missouri with Fremont and Hunter, was in Bank's Red River campaign. He went early in 1864 with Sherman to Meridian, Miss., and Selma, Ala. During the latter part of this year he returned to Connersville, resumed the practice of dentistry, remaining till 1872. He was a graduate of the Ohio College of Dental Surgery in 1870. A few years after his graduation he was connected with his Alma Mater as a teacher, and manifested rare ability in that work. In 1872 he practiced in San Francisco and Chicago. In January, 1873, he moved to Lexington, where he practiced up to the time of his death with great success.

Dr. Rawls possessed a very high sense of honor and maintained in a marked degree the dignity of his chosen profession. His attachments when formed were very strong, and his friendship, when fixed, was absolutely reliable. He was an earnest student on several lines of his profession and made very high attainments. His family will have the warmest sympathy of his numerous professional friends throughout the country.

The following action was taken by the profession of Lexington:

WHEREAS, Death has taken from our midst, in the full prime of life and usefulness, a talented and exemplary member of our profession, Dr. A. O. Rawls, whose career was crowded with vicissitudes; but whether as soldier, battling for his country's existence; student, preparing for the battle of life; professor, teaching others to be self-sustaining, or practitioner in his honorable calling, he was always a man of honor and probity, carrying himself so as to gain the respect of his comrades at arms and his brothers of the dental profession, and the esteem and good will of all who had the pleasure of his acquaintance. He was a charter member of the Kentucky Dental Association, of which he was twice elected president, and was a member of the State Board of Dental Examiners; for years was president of the Mississippi Valley and also Southern Dental Associations, and in the American Dental Association he was honored with office. In the presence of so sad a death it is meet that members of the dental profession in the city of Lexington take some formal action betokening their unaffected sorrow at the loss which they have sustained.

*Resolved*, By the dental surgeons here present that we deeply deplore the death of Dr. Albert O. Rawls, regarding it as a personal as well as a professional loss to us as well as to his large clientage and incidentally to the community. He was a valued citizen, and as a friend was faithful and true, a devoted husband, and indulgent father. He worked faithfully in his chosen field and his labors were crowned with success. To the younger members of his profession his course will serve as a stimulant to them to strive to reach the highest standard of excellence. He understood the demands upon him as a practitioner and citizen and responded fully to the requirements in every respect.

*Resolved*, That we tender to the bereaved family of the deceased our heartfelt sympathy in their present grief and that a copy of these resolutions be presented to them and also published in the city papers and dental journals.

DR. T. D. KELLEY, Chairman.
DR H. L. HARLAN, Secretary.

### William Henry Eames, D.D.S.

Dr. William Henry Eames died in St. Louis, at the residence of his daughter, Dr. Emma Eames-Chase, on March 29, after a short illness.

He was born in Auburn, N. Y., August 23, 1828. His father, George Eames, soon after the birth of this son, removed to Oneida county, N. Y., where his father, James Eames, with his large family had settled in 1794. They were progressive people, taking an active part in all public affairs, and particularly interested in educational advancement. The active interest, untiring zeal and devotion with which Dr. Eames labored for the advancement of his profession, in the college, in the journals, and in societies was a direct continuation of the spirit with which his father and grandfather before him had worked for the intellectual advancement of the rising generation. He was educated in the public schools and at the Clinton Academy, in Oneida county. After teaching school several years he commenced the study of medicine with Dr. Porter, an eminent English physician, and went to Ann Arbor to complete his medical studies at the University of Michigan. While there he decided to study dentistry, and entered the Ohio College of Dental Surgery, graduating in 1583. He returned to Ann Arbor and formed a partnership with his preceptor, Dr. Porter.

He was married in 1855 to Laura M. Scofield, of Clinton, Michigan, and removed to Lebanon, Tenn., in 1857, remaining there until 1862, when, owing to the disturbed condition of the south, he removed to St. Louis, Mo. He at once became a member of the St. Louis Dental Society, and always took an active and prominent part in the profession in St. Louis. It was at a meeting of the Society at his residence that the subject of organizing a dental college in St. Louis was introduced. There were present at this meeting Drs. Judd, McKellops, Forbes, Peebles and others, and from its first inception he was an active worker, continuing through many years to the very night before he was stricken down. He was one of the founders of the *Missouri Dental Journal,* and for many years its editor; and later, editor of the *Archives of Dentistry*, which succeeded it.

Dr. Eames was an active member of the American Dental Association, the Missouri State Dental Association, and the St. Louis Dental Society; the last two of which he has been President at different times. He had also served as President of the Association of Dental College Faculties, and was an honorary member of the Illinois, Iowa, and other State and district dental societies.

It is not necessary to speak here of the genial nature and the many admirable qualities of our departed brother; these are known to all who ever had the pleasure of his acquaintance, and will be referred to as pleasant recollections until all have followed him to the other side.

The doctor leaves a wife and seven grown children; two sons and five daughters.—*The Dental Review.* A. H. Fuller.

---

### RESOLUTIONS OF RESPECT.

The following resolutions on the death of Prof. W. H. Eames, of St. Louis, were adopted at the regular meeting of the Chicago Dental Society, April 3, 1894.

Whereas: It has come to the knowledge of the members of the Chicago Dental Society that the dental profession is called to mourn the loss of one of its most distinguished and honored members;

*Therefore be it resolved*, That, in the decease of Prof. Eames, the world has lost one of its foremost educators, his family a devoted husband and father, and the profession a sincere earnest and useful devotee. It is fitting that this society should pay its tribute of respect to one who has so long and honorably filled positions before the world, and we mourn his death as a personal loss to each and all of us.

*Be it further resolved,* That a copy of the above be transmitted to the family of the deceased, and a further copy be sent to the journals for publication.

A. W. Harlan.
Truman W. Brophy.
Edgar D. Swain.

